# Declining Rates in Male Circumcision amidst Increasing Evidence of its Public Health Benefit

**DOI:** 10.1371/journal.pone.0000861

**Published:** 2007-09-12

**Authors:** Zohar Mor, Charlotte K. Kent, Robert P. Kohn, Jeffrey D. Klausner

**Affiliations:** 1 Hubert H. Humphrey Fellowship Program, Rollins School of Public Health, Emory University, Atlanta, Georgia, United States of America; 2 Sexually Transmitted Disease Prevention and Control Section, San Francisco Department of Public Health, California, United States of America; University of Stellenbosch, South Africa

## Abstract

**Background:**

Recent experimental evidence has demonstrated the benefits of male circumcision for the prevention of human immunodeficiency virus (HIV) infection. Studies have also shown that male circumcision is cost-effective and reduces the risk for certain ulcerative sexually transmitted diseases (STDs). The epidemiology of male circumcision in the United States is poorly studied and most prior reports were limited by self-reported measures. The study objective was to describe male circumcision trends among men attending the San Francisco municipal STD clinic, and to correlate the findings with HIV, syphilis and sexual orientation.

**Methods and Findings:**

A cross sectional study was performed by reviewing all electronic records of males attending the San Francisco municipal STD clinic between 1996 and 2005. The prevalence of circumcision over time and by subpopulation such as race/ethnicity and sexual orientation were measured. The findings were further correlated with the presence of syphilis and HIV infection. Circumcision status was determined by physical examination and disease status by clinical evaluation with laboratory confirmation. Among 58,598 male patients, 32,613 (55.7%, 95% Confidence Interval (CI) 55.2–56.1) were circumcised. Male circumcision varied significantly by decade of birth (increasing between 1920 and 1950 and declining overall since the 1960's), race/ethnicity (Black: 62.2%, 95% CI 61.2–63.2, White: 60.0%, 95% CI 59.46–60.5, Asian Pacific Islander: 48.2%, 46.9–49.5 95% CI, and Hispanic: 42.2%, 95% CI 41.3–43.1), and sexual orientation (gay/bisexual: 73.0%, 95% CI 72.6–73.4; heterosexual: 66.0%, 65.5–66.5). Male circumcision may have been modestly protective against syphilis in HIV-uninfected heterosexual men (PR 0.92, 95% C.I. 0.83–1.02, P = 0.06).

**Conclusions:**

Male circumcision was common among men seeking STD services in San Francisco but has declined substantially in recent decades. Male circumcision rates differed by race/ethnicity and sexual orientation. Given recent studies suggesting the public health benefits of male circumcision, a reconsideration of national male circumcision policy is needed to respond to current trends.

## Introduction

In North America, male circumcision has been promoted for more than a century by the medical community and is an accepted social norm [1]. Based on a small U.S. probability sample (n = 1410) using self-report of circumcision status conducted in 1992, it was estimated that 77% of males born in the United States (U.S.) are circumcised [2]. Even though male circumcision is one of the most common operations performed in the U.S. [3] it has become highly controversial. Advocates who oppose male circumcision have challenged its medical benefits, questioned its ethical underpinnings, and highlighted its potential associated risks [1], [4].

The first report suggesting male circumcision was protective against HIV infection was published in 1986 [5]. Many subsequent studies have demonstrated that male circumcision reduces susceptibility to HIV infection with a protective effect ranging between 23% and 88% [6], [7]. Three recently completed randomized-controlled trials have confirmed the protective effect of male circumcision demonstrating a reduced risk of HIV acquisition between 48–60%. All trials were discontinued by the ethical boards, as significant results in HIV reduction were achieved in the interim analyses [8]–[10]. Following those encouraging findings, male circumcision was recently recommended by the WHO and the UNAIDS [11]. Other studies have suggested that male circumcision was associated with lower risk of genital ulcerative disease, such as chancroid and syphilis [12], [13], decreased risks for urinary tract infections [14] and for penile cancer [15], as well as it being a cost-effective procedure [16], [17].

Given the recent interest in male circumcision and its demonstrated benefits, further understanding of the trends and distribution of male circumcision in the U.S. is critical. We describe the epidemiology of male circumcision based on physical examination among male patients visiting the San Francisco municipal STD clinic during the recent decade.

## Methods

We reviewed electronic medical records among male patients visiting the San Francisco municipal STD clinic between January 1, 1996 and December 31, 2005.

Following clinic face-to-face registration to collect self-reported demographic (including race/ethnicity) and behavioral information, clinicians performed physical examination of the genitalia among all male patients. Clinicians recorded penile circumcision status in the medical records as “Yes” or “No.” The remainder of the examination, diagnostic testing and treatment was performed according to standard clinic protocols.

In order to examine the relationship between circumcision status, HIV and ulcerative infection, such as syphilis, prevalence ratios (PR) were computed by comparing infection status in visits of circumcised and non-circumcised men. Each of the infections was counted only once, e.g, patient-visits in HIV-infected patients were restricted to one count, even if multiple visits were made. For birth cohort trend analyses of circumcision we compared unique patient records excluding those of persons with repeat visits. Using standard methods, 95% Exact Confidence Intervals (CI) were calculated (Stata 5.0, StataCorp, LP, Texas, U.S.A.) Proportions were compared by Chi-square analysis. As these were de-identified medical records undergoing retrospective analyses, this study was considered exempt from human subjects considerations in accordance with the Code of Federal Regulations, Title 45.

## Results

Between January 1, 1996 and December 31, 2005, 58,598 male patients were examined at the San Francisco municipal STD clinic. During that period, these men made 154,177 clinic visits, with an average of 2.6 visits per patient. The majority of male patients were white (54.1%), born before 1970 (52.3%), and 35.5% identified as gay/bisexual (Table 1). Fifty-one percent of patient visits were by gay or bisexual men. Among patients, 32,613 (55.7%, 95% CI 55.2–56.1) were circumcised. Circumcision status varied by race/ethnicity (Table 1): the highest proportion was 62.2% among Blacks to the lowest proportion of 42.2% among Hispanics (P<.001); and by sexual orientation 66.0% (95% CI 65.5–66.5) among heterosexual men and 73.0% (95% CI 72.6–73.4) among gay/bisexual men (P<.001).

**Table 1:** Selected demographic characteristics of male patients: San Francisco municipal STD clinic, 1996–2005.

Characteristic	Patients	% of the total	Circumcised
	N		N (%) [95% CI]
Race/ethnicity			
Black	9,214	15.8	5,731 (62.2) [61.2–63.2]
White	31,685	54.1	18,996 (60.0) [59.4–60.5]
Asian/Pacific Islander	5,876	10.0	2,834 (48.2) [46.9–49.5]
Hispanic	10,980	18.7	4,634 (42.2) [41.3–43.1]
Missing, unknown	843	1.4	397 (47.1) [43.7–50.5]
Birth Decade			
1900–1920	178	0.3	28 (15.7) [10.7–21.9]
1930	557	1.0	262 (47.0) [42.8–51.3]
1940	2,696	4.6	1,421 (52.7) [50.8–54.6]
1950	8,281	14.1	4,844 (58.5) [57.4–59.6]
1960	18,981	32.4	11,275 (59.4) [58.7–60.1]
1970	21,837	37.3	12,098 (55.4) [54.7–56.1]
1980–1990	5,606	9.6	2,418 (43.1) [41.8–44.5]
Missing, unknown	462	0.7	255 (55.2) [50.5–59.8]
Sexual Orientation			
Gay, bisexual	20,832	35.5	12,577 (60.4) [59.7–61.0]
Heterosexual	33,867	57.8	18,352 (54.2) [53.7–54.7]
Missing, unknown	3,899	6.7	2,253 (57.8) [56.2–59.3]
Total	58,598		32,613 (55.7) [55.2–56.1]

**Figure 1:**
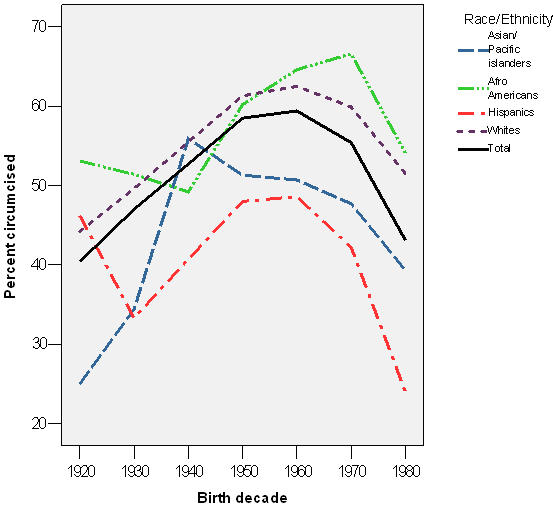
Trends in circumcision proportion of male patients by birth decade and race/ethnicity San Francisco municipal STD clinic, 1920–1980.

By decade of birth, we observed increasing rates of circumcision among men born between 1920 and 1950, and then an average 33.4% decline in rates of circumcision among those born after the 1960's (Figure 1). Those trends were similar across all racial/ethnic groups.

Table 2 shows the proportion of visits by circumcised men at the San Francisco municipal STD clinic from 1996 through 2005 by sexual orientation, syphilis and HIV infection status. There was a trend towards a protective effect of circumcision for syphilis infection in heterosexual HIV-uninfected men and in a lesser extent in HIV-infected men. Among gay/bisexual men, no such protective effect was seen and also no association was found between circumcision status and HIV infection (71.1% circumcised *versus* 72.2%, PR = 0.97, 95% CI 0.90-1.0, P = 0.52).

**Table 2:** Percent circumcised in those with and without syphilis infection by HIV status and sexual orientation, as determined during male patient visits, San Francisco municipal STD clinic, 1996–2005.

Sexual orientation	Syphilis infection	HIV-infected		HIV -uninfected	
		Circumcised % (n/N)	PR* (95% CI)	Circumcised % (n/N)	PR (95% CI)
Heterosexual	Yes	62.5 (10/16)	0.85 (0.40–1.56)	66.7 (384/576)	0.92 (0.83–1.02)
	No	73.8 (1,050/1,423)	Ref.	72.4 (36,290/50,128)	Ref.
Gay/ bisexual	Yes	75.8 (214/282)	1.0 (0.87–1.15)	72.7 (384/528)	0.98 (0.88–1.08)
	No	75.4 (15,910/21,090)	Ref.	74.6 (34,210/45,869)	Ref.

* PR = Prevalence ratio of circumcision status by syphilis infection (Yes/No)

## Discussion

A majority (55.6%) of male patients attending the San Francisco municipal STD clinic between 1996 and 2005 were circumcised. African-American and White men were more commonly circumcised than Hispanic men and men of Asian/ Pacific Islander descent. A steady and substantial decline in circumcision rates was observed beginning in men born after 1960 across all racial/ethnic groups. There was a trend towards a protective effect of male circumcision in heterosexual men for syphilis infection.

The strengths of this study include a very large sample population that visited the clinic within a decade and could be representative of current male residents throughout the U.S. At least 85% of men seen at the San Francisco municipal STD Clinic during the study period were San Francisco residents. Census data in San Francisco suggest that most county residents were born in the U.S. (63%) but only 35% were born in California suggesting that most STD clinic patients were born outside of California and might represent other U.S. regions [18]. Therefore, the characteristics of our study population by race/ethnicity might be comparable to those of men currently living in the U.S.

In addition, circumcision status was determined by physical examination by trained clinicians and both syphilis and HIV-infection status were determined by clinical testing with laboratory confirmation.

Male circumcision is an accepted procedure in Jewish and Muslim-dominated countries and also in other areas, like North America and Korea [19]. The decision to circumcise is usually made by parents, and stems from religious practices, cultural and social factors, and health beliefs. While we observed declines in circumcision among younger men across all racial/ethnic groups, we found that both Black and White men were circumcised more often in comparison with other racial/ethnic groups, supporting the role that socio-cultural factors play in circumcision practices

Most prior studies correlating male circumcision and the prevalence of HIV infection were ecological or cross-sectional and recent clinical trials have been performed in Africa, where the HIV epidemic is generalized and the mode of transmission is mainly heterosexual. However, the relationship between circumcision status and STDs or HIV infection in gay or bisexual men has been poorly studied. A cross-sectional study in the early 1990's conducted in the U.S. [20] found that uncircumcised gay men were two-fold more likely to be infected with HIV. A recent study [21] found similar elevated risk of HIV acquisition among uncircumcised gay men, but did not correlate specific sexual practices (i.e., insertive or receptive anal sex) and circumcision status with HIV acquisition. Our study included a very large number of visits made by gay or bisexual men (nearly 80,000 visits). Our findings, showing no significant differences between circumcision status and the risk of HIV or syphilis infection, are consistent with the importance of non-penile, rectal acquisition of those infections (or oral infection in syphilis) among gay men in the U.S. [22], rather than penile acquisition as in heterosexual intercourse, during which the foreskin may be exposed to HIV infection. Because large proportions of gay men practice both insertive and receptive anal intercourse [23], the ability to differentiate between different risks for HIV infection associated with sexual practices *versus* circumcision status is limited. Consequently, gay men and other men who have sex with men might not gain the same level of protection from circumcision as heterosexual men.

In this study, male circumcision was found to be consistent with protection against syphilis infection in heterosexual men, similar to prior reports [12], [13]. That finding in our study supports the validity of our measurement of circumcision status as it corroborates prior epidemiologic evidence and the current biological understanding of the foreskin. Foreskin, after exposure during erection, provides a warm, moist and supportive environment for infectious agents possibly prolonging those pathogens' survival [24]; it also may cover ulcers or sores, which consequently may be responsible for delayed diagnosis and treatment [25]. In addition, the mucosal aspect of the foreskin is poorly keratinized and contains a high density of Langerhans cells, susceptible target cells for viral infections like HIV that are suspected to facilitate the delivery of pathogens into regional lymph nodes and further to the blood stream [26].

Nevertheless, policy makers should be aware that as circumcision is perceived by the public to be protective against HIV acquisition, it may encourage behavioral disinhibition and should be included within a comprehensive package of sexual health promotion. Increases in high-risk sexual behavior by circumcised men could compensate for the benefits of circumcision and reduce its overall impact

Limitations of our study should be addressed. First, the study design was a retrospective cross-sectional analysis, for which it is usually difficult to ascertain the temporal sequence of exposure and outcome. Because most U.S. males are circumcised during infancy, it is unlikely that men in our study acquired STDs or HIV infection prior to their circumcision. While a very large sample, the true generalizability of our findings outside of men attending that clinic is unknown. The large sample size, however, may allow our results to be compared to other studies in the U.S, and findings from subpopulations in our clinic might be similar to comparable subpopulations in the U.S. It is also important to indicate that some population composition and behavioral changes may have occurred during this decade in San Francisco. However, our main results were by decade of birth and stratified by race/ethnicity so that should have controlled for differences in the underlying population over time. Secondly, behavioral attributes for the study participants were not available and neither were characteristics of repeat visits, therefore a limited analysis was presented.

An important strength of our study was that circumcision status was determined by clinical examination reducing the likelihood of misclassification. Several studies have found that objective penile physical examination yield more accurate results regarding circumcision status than self-report [2], [6], [27].

In conclusion, while a majority of men attending the San Francisco STD clinic were circumcised, there were large and steady declines in circumcision across all racial/ethnic groups since 1960. There were significant differences by racial/ethnic groups suggesting important socio-cultural factors related to decisions to circumcise newborn males. Given the recent evidence demonstrating the substantial potential public health benefit of male circumcision and our observed declines in circumcision rates, national organizations that promote circumcision policy should review current practice guidelines in responding to those trends.
